# Terapêutica da ICFER: Evidência Randomizada e Mundo Real

**DOI:** 10.36660/abc.20260588

**Published:** 2026-09-08

**Affiliations:** 1 Hospital Meridional de Cariacica Cariacia ES Brasil Hospital Meridional de Cariacica, Cariacia, ES – Brasil

**Keywords:** Insuficiência Cardíaca, Insuficiência Cardíaca Sistólica, Prática Clínica Baseada em Evidências

## Resumo

A insuficiência cardíaca com fração de ejeção reduzida (ICFER) apresentou importante avanço terapêutico com a consolidação dos quatro pilares farmacológicos modificadores do prognóstico. A utilização abrangente dessas terapias se associa a expressiva redução de morte cardiovascular e hospitalizações por insuficiência cardíaca, sustentando estratégias contemporâneas de rápida introdução e posterior otimização até as doses-alvo ou máximas toleradas. Nesse contexto, o sacubitril/valsartana demonstrou benefício prognóstico no PARADIGM-HF e maior redução do NT-proBNP no PIONEER-HF. Entretanto, resultados distintos foram observados em outros cenários, como no PARADISE-MI e no LIFE, contribuindo para delimitar as populações nas quais seu benefício foi demonstrado. O estudo publicado nesta edição dos Arquivos Brasileiros de Cardiologia avaliou, em uma coorte observacional de mundo real, pacientes com ICFER tratados com doses máximas de sacubitril/valsartana, enalapril ou losartana. Em comparação ao grupo tratado com enalapril ou losartana, o sacubitril/valsartana não se associou à redução estatisticamente significativa de hospitalização ou mortalidade, embora tenha se associado à melhora da classe funcional. Esses achados devem ser interpretados considerando limitações metodológicas, como desenho observacional, ausência de randomização, número restrito de eventos e heterogeneidade do grupo comparador. Estudos de mundo real ampliam a compreensão sobre efetividade, aplicabilidade e implementação das terapias, devendo ser analisados em conjunto com as evidências consolidadas dos ensaios clínicos randomizados.

A insuficiência cardíaca (IC) é uma síndrome complexa e grave, associada a elevadas taxas de hospitalização e mortalidade. Na ICFER, grandes ensaios clínicos estabeleceram quatro pilares farmacológicos modificadores do prognóstico: betabloqueadores, que atenuam a ativação do sistema nervoso simpático; inibição do sistema renina-angiotensina, através do uso do inibidor do receptor da angiotensina e da neprilisina (ARNI), inibidor da enzima conversora da angiotensina (IECA) ou bloqueador do receptor da angiotensina (BRA); antagonistas dos receptores mineralocorticoides (ARM) e inibidores do cotransportador sódio-glicose tipo 2 (iSGLT2).^1,2^

Uma análise comparativa de três ensaios clínicos estimou que o uso conjunto dessas terapias, em comparação ao tratamento convencional com IECA ou BRA associado a betabloqueador, poderia reduzir o risco de morte cardiovascular ou hospitalização por IC em aproximadamente 62% (HR: 0,38; IC 95%: 0,30–0,47).³ Embora se trate de uma estimativa derivada de comparações entre estudos, e não de um ensaio clínico que tenha randomizado diretamente as duas estratégias, seus resultados podem respaldar a utilização abrangente da terapêutica quádrupla, em consonância com estratégias contemporâneas que priorizam a rápida introdução dos quatro pilares, seguida de otimização até as doses-alvo ou máximas toleradas.^4,5^

Nesse arsenal terapêutico, pautado no estudo PARADIGM-HF, o sacubitril/valsartana demonstrou redução significativa de mortalidade cardiovascular e hospitalizações por IC em comparação ao enalapril.^6^ No PIONEER-HF, sua introdução após estabilização clínica de pacientes hospitalizados por IC aguda promoveu maior redução do NT-proBNP do que o enalapril, embora o estudo não tenha sido dimensionado para estabelecer benefício definitivo sobre mortalidade.^7^ Em outros contextos clínicos, os resultados foram distintos. No PARADISE-MI, realizado após infarto agudo do miocárdio em pacientes com disfunção ventricular esquerda e/ou congestão pulmonar, o sacubitril/valsartana não reduziu significativamente o desfecho primário em comparação ao ramipril.^8^ Já no LIFE, em pacientes com ICFER avançada, não houve diferença significativa na redução do NT-proBNP em relação à valsartana.^9^ Em conjunto, essas evidências não contradizem os achados do PARADIGM-HF, mas contribuem para delimitar os contextos clínicos nos quais o benefício do ARNI foi demonstrado.

A questão se torna particularmente relevante na era da terapia quádrupla. À medida que o tratamento de base reduz o risco residual, as diferenças absolutas potencialmente atribuíveis a uma intervenção isolada podem diminuir, exigindo estudos maiores e maior número de eventos para demonstrar benefícios incrementais. Contudo, não há como afirmar que a utilização de novas drogas como iSGLT2 elimine o benefício do ARNI, uma vez que essa hipótese ainda não foi testada diretamente em ensaio clínico randomizado.

É nesse contexto que se insere o estudo publicado nesta edição dos Arquivos Brasileiros de Cardiologia.^10^ Em uma coorte observacional, os autores avaliaram 254 pacientes com ICFER tratados com sacubitril/valsartana versus enalapril ou losartana em doses alvo. Durante mediana de seguimento de 23,9 meses, ocorreram 70 eventos do desfecho primário. Após o ajuste, embora tenha sido documentada melhora funcional significativa avaliada pela classe funcional da New York Heart Association, não foi identificada associação estatisticamente significativa entre sacubitril/valsartana e o desfecho combinado de hospitalização ou morte (OR: 0,65; IC 95%: 0,35–1,20; p=0,165). Alguns aspectos metodológicos devem ser considerados na interpretação dos achados. O desenho observacional, com componentes prospectivo e retrospectivo, o número limitado de eventos e a amplitude dos intervalos de confiança podem restringir a precisão das estimativas e desta forma não concluir equivalência entre as estratégias. Além disso, a análise conjunta de enalapril e losartana introduz heterogeneidade no grupo comparador e pode limitar inferências específicas para cada fármaco. Por fim, a classificação de doses equivalentes de losartana merece ser interpretada com cautela, uma vez que o estudo HEAAL avaliou 150 mg/dia como estratégia de alta dose e demonstrou menor risco de morte ou hospitalização por IC com a dose mais elevada. Essas limitações, em grande parte reconhecidas pelos próprios autores, não reduzem a relevância do estudo, mas apontam cautela na generalização de suas conclusões.^1,2,11^

O trabalho também suscita uma discussão pertinente à realidade brasileira: a relação entre eficácia, efetividade, custo e acesso. Uma revisão sistemática recente de estudos econômicos brasileiros classificou o sacubitril/valsartana como custo-efetivo na perspectiva do Sistema Único de Saúde, mas destacou que muitas tecnologias foram avaliadas em apenas um estudo, limitando a robustez das conclusões.^12,13^

A principal contribuição desta coorte talvez não seja contestar o benefício individual demonstrado em ensaio clínico randomizado, mas mostrar a complexidade de avaliar uma intervenção isolada em um cenário terapêutico mais contemporâneo. Estudos de mundo real complementam os ensaios clínicos ao avaliar efetividade, implementação e aplicabilidade em populações menos selecionadas; seus achados são mais bem compreendidos quando analisados em conjunto com as evidências consolidadas provenientes de ensaios clínicos randomizados.

O desafio atual da ICFER permanece garantir a introdução precoce, abrangente, sustentada e economicamente viável das terapias comprovadamente eficazes, para que os avanços dos ensaios clínicos se traduzam em benefícios concretos e equitativos para os pacientes brasileiros.
