# Supplementary material for: Terapêutica da ICFER: Evidência Randomizada e Mundo Real
Source: Arq Bras Cardiol. 2026 Sep 8;123(7):e20260588. [Article in Portuguese] doi: 10.36660/abc.20260588 (PMC13668449; doi:10.36660/abc.20260588)
Supplement: Translation

# Translation: HFREF Therapy: Randomized Evidence and Real-World Perspectives

**DOI:** 10.36660/abc.20260588i

**Affiliations:** 1 Cariacia ES Brazil Hospital Meridional de Cariacica, Cariacia, ES – Brazil

**Keywords:** Heart Failure, Systolic Heart Failure, Evidence-Based Practice

## Abstract

Heart failure with reduced ejection fraction (HFrEF) has seen significant therapeutic advances with the consolidation of the four pharmacological pillars that modify prognosis. The widespread use of these therapies is associated with a substantial reduction in cardiovascular death and hospitalizations for heart failure, supporting contemporary strategies for rapid introduction and subsequent optimization to target or maximum tolerated doses. In this context, sacubitril/valsartan demonstrated prognostic benefit in PARADIGM-HF and a greater reduction in NT-proBNP in PIONEER-HF. However, different results were observed in other settings, such as PARADISE-MI and LIFE, helping to define the populations in which its benefit was demonstrated. The study published in this issue of the *Arquivos Brasileiros de Cardiologia* evaluated, in a real-world observational cohort, patients with HFrEF treated with maximum doses of sacubitril/valsartan, enalapril, or losartan. Compared to the group treated with enalapril or losartan, sacubitril/valsartan was not associated with a statistically significant reduction in hospitalization or mortality, although it was associated with an improvement in functional class. These findings should be interpreted considering methodological limitations, such as observational design, lack of randomization, limited number of events, and heterogeneity of the comparator group. Real-world studies broaden the understanding of the effectiveness, applicability, and implementation of therapies and should be analyzed in conjunction with the consolidated evidence from randomized clinical trials.

Heart failure (HF) is a complex and serious syndrome associated with high hospitalization and mortality rates. In heart failure with reduced ejection fraction (HFrEF), large clinical trials have established four pharmacological pillars that modify the prognosis: beta-blockers, which attenuate the activation of the sympathetic nervous system; inhibition of the renin-angiotensin system, through the use of angiotensin receptor and neprilysin inhibitors (ARNIs), angiotensin-converting enzyme inhibitors (ACEIs), or angiotensin receptor blockers (ARBs); mineralocorticoid receptor antagonists (MRAs); and sodium-glucose cotransporter type 2 inhibitors (SGLT2i).^1,2^

A comparative analysis of three clinical trials estimated that the combined use of these therapies, compared to conventional treatment with ACEI or ARB associated with a beta-blocker, could reduce the risk of cardiovascular death or hospitalization for HF by approximately 62% (HR: 0.38; 95% CI: 0.30–0.47).³ Although this estimate is derived from comparisons between studies, and not from a clinical trial that directly randomized the two strategies, its results may support the widespread use of quadruple therapy, in line with contemporary strategies that prioritize the rapid introduction of the four pillars, followed by optimization to target or maximum tolerated doses.^4,5^

In this therapeutic arsenal, based on the PARADIGM-HF study, sacubitril/valsartan demonstrated a significant reduction in cardiovascular mortality and hospitalizations for HF compared to enalapril.^6^ In the PIONEER-HF study, its introduction after clinical stabilization of patients hospitalized for acute HF promoted a greater reduction in NT-proBNP than enalapril, although the study was not designed to establish a definitive benefit on mortality.^7^ In other clinical contexts, the results were different. In the PARADISE-MI study, conducted after acute myocardial infarction in patients with left ventricular dysfunction and/or pulmonary congestion, sacubitril/valsartan did not significantly reduce the primary outcome compared to ramipril.^8^ In the LIFE study, in patients with advanced HFrEF, there was no significant difference in the reduction of NT-proBNP compared to valsartan.^9^ Taken together, this evidence does not contradict the findings of PARADIGM-HF, but it does help to define the clinical contexts in which the benefit of ARNI has been demonstrated.

The issue becomes particularly relevant in the era of quadruple therapy. As background treatment reduces residual risk, the absolute differences potentially attributable to a single intervention may diminish, requiring larger studies and a greater number of events to demonstrate incremental benefits. However, it cannot be stated that the use of new drugs such as SGLT2 inhibitors eliminates the benefit of ARNI, since this hypothesis has not yet been directly tested in a randomized clinical trial.

It is in this context that the study published in this issue of the *Arquivos Brasileiros de Cardiologia* is situated.^10^ In an observational cohort, the authors evaluated 254 patients with HFrEF treated with sacubitril/valsartan versus enalapril or losartan at target doses. During a median follow-up of 23.9 months, 70 primary outcome events occurred. After adjustment, although significant functional improvement was documented as assessed by the New York Heart Association functional class, no statistically significant association was identified between sacubitril/valsartan and the combined outcome of hospitalization or death (OR: 0.65; 95% CI: 0.35–1.20; p=0.165). Several methodological aspects should be considered when interpreting the findings. The observational design, with prospective and retrospective components, the limited number of events, and the width of the confidence intervals may restrict the precision of the estimates and thus prevent concluding equivalence between the strategies. Furthermore, the combined analysis of enalapril and losartan introduces heterogeneity in the comparator group and may limit specific inferences for each drug. Finally, the classification of equivalent doses of losartan should be interpreted with caution, since the HEAAL study evaluated 150 mg/day as a high-dose strategy and demonstrated a lower risk of death or hospitalization for heart failure with the higher dose. These limitations, largely acknowledged by the authors themselves, do not diminish the relevance of the study, but point to caution in generalizing its conclusions.^1,2,11^

The work also raises a discussion relevant to the Brazilian reality: the relationship between efficacy, effectiveness, cost, and access. A recent systematic review of Brazilian economic studies classified sacubitril/valsartan as cost-effective from the perspective of the Unified Health System but highlighted that many technologies were evaluated in only one study, limiting the robustness of the conclusions.^12,13^

The main contribution of this cohort may not be to challenge the individual benefit demonstrated in randomized clinical trials, but to show the complexity of evaluating an isolated intervention in a more contemporary therapeutic setting. Real-world studies complement clinical trials by evaluating effectiveness, implementation, and applicability in less selected populations; their findings are better understood when analyzed in conjunction with the consolidated evidence from randomized clinical trials.

The current challenge for HFREF remains ensuring the early, comprehensive, sustained, and economically viable introduction of proven effective therapies, so that advances in clinical trials translate into concrete and equitable benefits for Brazilian patients.
